# Knowledge, attitude and practice towards tuberculosis in Gambia: a nation-wide cross-sectional survey

**DOI:** 10.1186/s12889-020-09685-3

**Published:** 2020-10-17

**Authors:** Adedapo Olufemi Bashorun, Christopher Linda, Semeeh Omoleke, Lindsay Kendall, Simon D. Donkor, Ma-Ansu Kinteh, Baba Danso, Lamin Leigh, Sheriff Kandeh, Umberto D’Alessandro, Ifedayo Morayo O. Adetifa

**Affiliations:** 1 Disease Control and Elimination Theme, Medical Research Council Unit The Gambia at London School of Hygiene and Tropical Medicine, P.O. Box 273, Banjul, The Gambia; 2 Department of Infectious Diseases Epidemiology, London School of Hygiene and Tropical Medicine, London, UK

**Keywords:** Tuberculosis, Knowledge, Attitude, Practice, Stigma, Prevalence survey, Africa

## Abstract

**Background:**

Early diagnosis and treatment of tuberculosis (TB) are the mainstay of global and national TB control efforts. However, the gap between expected and reported cases persists for various reasons attributable to the TB services and care-seeking sides of the TB care cascade. Understanding individual and collective perspectives of knowledge, attitudes, beliefs and other social circumstances around TB can inform an evidence-based approach in engaging communities and enhance their participation in TB case detection and treatment.

**Methods:**

The study was conducted during the Gambian survey of TB prevalence. This was a nationwide cross-sectional multistage cluster survey with 43,100 participants aged ≥15 years in 80 clusters. The study sample, a random selection of 10% of the survey population within each cluster responded to a semi-structured questionnaire administered by trained fieldworkers to assess the knowledge, attitudes and practice of the participants towards TB. Overall knowledge, attitude and practice scores were dichotomised using the computed mean scores and analysed using descriptive, univariable and multivariable logistic regression.

**Results:**

All targeted participants (4309) were interviewed. Majority were females 2553 (59.2%), married 2614 (60.7%), had some form of education 2457 (57%), and were unemployed 2368 (55%). Although 3617 (83.9%) of the participants had heard about TB, only 2883 (66.9%) were considered to have good knowledge of TB. Overall 3320 (77%) had unfavourable attitudes towards TB, including 1896 (44%) who indicated a preference for staying away from persons with TB rather than helping them. However, 3607(83.7%) appeared to have the appropriate health-seeking behaviours with regard to TB as 4157 (96.5%) of them were willing to go to the health facility if they had symptoms suggestive of TB.

**Conclusions:**

About 3 in 10 Gambians had poor knowledge on TB, and significant stigma towards TB and persons with TB persists. Interventions to improve TB knowledge and address stigma are required as part of efforts to reduce the burden of undiagnosed TB in the country.

**Supplementary information:**

**Supplementary information** accompanies this paper at 10.1186/s12889-020-09685-3.

## Background

With the implementation of the End TB strategy, the global incidence of tuberculosis (TB) fell at ~ 2% annually from 2000 to 2018, but this decline was too slow to attain global TB control targets – 20% reduction in global incidence between 2015 and 2018, 80% reduction in TB incidence and 90% reduction in TB deaths by 2030 in comparison to 2015 [1, 2]. This situation is replicated in The Gambia where the decline in TB incidence has stagnated - 175 per 100,000 population in 2012 [3] to 174 per 100,000 population in 2018 [1]. Worldwide, TB continues to be responsible for the most deaths attributable to a single infectious disease [1].

Detecting and treating primary and secondary TB cases with the aim of increasing TB treatment coverage is a major component of the End TB Strategy [1]. However, despite ongoing efforts, there remains a significant gap between the number of notified and incident cases. This is reflected in the significant burden of undiagnosed TB cases found in the community during TB prevalence surveys in The Gambia [3] and elsewhere in Africa [4–10]. Addressing the gap in TB case finding requires an understanding of who is missed and how to decrease delays in TB case detection [11]. Lack of high quality TB services may be one factor [11]. However, delay in people seeking healthcare, despite adequate service provision, due to a failure to recognize the significance of a given symptom complex may be another important element [11, 12]. Delays in case detection, notification and treatment are known to be associated with poor perception of and confidence in available health care facilities and staff; stigma and poor knowledge of TB including misconceptions regarding transmission and prevention [2, 13–20]. Care seeking behaviour has been shown to be influenced by knowledge about the symptoms and signs of TB [20–23]. Thus, interventions to address these societal or community-level factors are required to improve or contribute to TB case detection and treatment success. It is for this reason that effective community engagement including advocacy, communication and social mobilisation activities (ACSM) is an essential component of the 2nd pillar of the World Health Organisation’s End TB strategy [1].

In The Gambia, the National Leprosy and Tuberculosis Control Programme’s (NLTP) strategic plan includes ACSM which it implements principally to sensitise communities to enhance their participation in TB case finding [24]. A better understanding of community-level knowledge, attitude and practices (KAP) related to TB can help the NLTP design and implement evidence-driven ACSM activities as part of its mandate to control TB in The Gambia. The evidence-driven ACSM activities are key part of NLTP’s acceleration of quality health services and universal coverage policy [25]. Therefore, as part of the first TB prevalence survey in the Gambia, [3] we assessed the knowledge, attitude and practice (KAP) related to TB in a nationally representative population.

## Methods

### Study design

This was a cross-sectional survey conducted alongside the Gambian Survey of TB Prevalence (GAMSTEP) – the parent study that was a nationwide multistage cluster survey conducted in 80 clusters allocated to regions of the country in proportion to population size [3].

### Study setting

The study was conducted from 2011 to 2013 in The Gambia which is the smallest mainland country in Africa with a population of about 1.9 million [26] and has had an average rate of growth of 2.8% per year over the last decade [27]. It is one of the most densely populated countries in Africa with an estimate of 177 people per square kilometre [27].

### Study participants

Eligible participants were those ≥15 years, resident in each cluster, spent at least one night in the household in the preceding 4 weeks and/or visitors who had arrived in the household 4 weeks or more before the survey [3]. On each survey day, the team chooses a starting number by taking the first number of the serial number on any random Gambian dalasis (currency) note after which all the tenth survey participants starting from this number were then selected for the KAP survey.

### Study variables

The main outcomes of interest were knowledge, attitude and practice of study participants while the exposure variables were areas of residence. Our general predictors were age, sex, marital status, occupational status and employment status.

### Data sources, measurement and analysis

Participants were interviewed in their own language by trained field workers using a pre-tested 29-item questionnaire (see additional file 1). The questionnaire was developed for this study based on the previous work in the country which identified gaps in attitude and knowledge [16, 28] and adapted content from a sister WHO-assited national TB prevalence survey in Ethiopia [4] and the WHO TB prevalence survey handbook [29]. Prior to the main study, the questionnaire was piloted and standardised as well as training on key words translations in the several spoken Gambian languages, so as to ensure consistency. And during the conduct of the study, there were several refresher training sessions.

We recorded responses to questions about socio-demographic characteristics, knowledge about TB, health care seeking behaviour, and stigma towards TB.

All data were double entered into a MySQL version 5.6.19 (Oracle, Redwood Shores, USA) relational database. Data analyses was done using Stata version 12.0 (Stata Corp., College Station, USA).

Overall, TB knowledge was assessed by 7 main questions about TB symptoms, TB transmission, ways to avoid getting TB, risk acquiring TB, whether TB can be cured and how, and cost of TB treatment within the country. Some of these questions had multiple answer options, thus the correct options (12 items) were used in the classification of the knowledge level. A score of one was given for each correct response and zero was given to incorrect and ‘do not know’ responses. Participants who had a score equal to and above the mean score of the study population were considered to have ‘good’ knowledge of TB and those with below the mean score were considered to have ‘poor’ knowledge of TB.

Similarly, we assessed attitude to TB via 4 questions: understanding that TB can affect anyone, a favourable reaction if found to have TB, who they will be willing to talk to if found to have TB, and desire to help people with TB. Using the mean score, participants were categorised as having ‘favourable’ or ‘unfavourable’ attitudes.

Practice or health seeking-behaviour was assessed with 2 questions: willingness to go to the health facility, and plan for timely visit to a health facility if respondents thought they had TB symptoms. Participants were considered to have ‘good’ practice or ‘poor’ practice using a mean score as a cut-off.

### Sample size

The TB prevalence survey (GAMSTEP) had a very large sample size of 55, 281. The target sample size for the KAP survey was 10% of all GAMSTEP participants and was chosen due to feasilibility, practicability and giving the unknown prevalence of the outcomes of interest. Thus, assuming 50% prevalence, the 10% of 55,281 would provide a > 80% power for a statistical meaningful deduction.

### Statistical analysis

Descriptive data were summarised and presented as frequencies and percentages (proportions). We tabulated categorical responses in proportions and assessed for associations with residence using the Pearson Chi-square test and assessed for differences in proportions using tests of equality of proportions. Associations between participant characteristics and each of the 3 outcome variables (TB knowledge, attitude and practice) were explored in univariable logistic regression analyses. A multivariable logistic regression analysis was performed including all the independent variables that showed significant association at a 5% level in the univariable analysis. We used a random-effects logistic regression model to account for clustering in the multistage sampling used for the parent study. Due to the exploratory nature of the analysis, no adjustment was made for multiple testing. There was < 1% missing data which was not corrected for as this did not alter the result.

### Ethical approval

This study was approved by the Joint Ethics Committee of the Gambian Government and Medical Research Council Unit, The Gambia, as part of the national TB survey. Written informed consent was obtained from all participants.

## Results

### Demographic characteristics

A total of 4309 of 43,100 participants in the main survey were interviewed. The mean age of the study population was 32.5 years (interquartile range, IQR 20–65 years). As shown in Table 1, the majority of our study population were females (59.2%), married (60.7%), unemployed (55%) and resided in rural areas (59.4%). A significant proportion of the population were illiterate (43%), and of Mandinka ethnicity (34.3%).

**Table 1:** Socio-demographic characteristics of the respondents

Characteristic	Rural – 2558 ***N*** (%)	Urban – 1751 ***N*** (%)	Total – 4309 ***N*** (%)
**Sex**			
Female	1568 (61.3)	985 (56.2)	2553 (59.2)
Male	990 (38.7)	766 (43.8)	1756 (40.8)
**Age distribution**			
15–34 years	1568 (61.3)	1167 (66.7)	2735 (63.5)
35–64 years	817 (31.9)	534 (30.5)	1351 (31.4)
65+ years	173 (6.8)	50 (2.8)	223 (5.1)
**Marital status**			
Married	1708 (66.7)	906 (51.7)	2614 (60.7)
Single	738 (28.9)	758 (43.3)	1496 (34.7)
Widow/widower	91 (3.6)	30 (1.7)	148 (3.4)
Separated/Divorced	21 (0.8)	57 (3.3)	51 (1.2)
**Educational status**			
Illiterate	1315 (51.4)	537 (30.7)	1852 (43.0)
Primary/Incomplete Secondary school	462 (18.1)	512 (29.2)	974 (22.6)
Read English/Arabic only	513 (20.0)	264 (15.1)	777 (18.0)
Complete Secondary school and above	268 (10.5)	438 (25.0)	706 (16.4)
**Occupational status**			
Unemployed	1416 (55.3)	952 (54.4)	2368 (55.0)
Employed	1140 (44.6)	794 (45.3)	1934 (44.9)
*Others	2 (0.1)	5 (0.3)	7 (0.1)
**Ethnicity**			
Mandinka	862 (33.7)	617 (35.3)	1479 (34.3)
Fula	656 (25.7)	398 (22.7)	1054 (24.5)
Wolof	392 (15.3)	216 (12.3)	608 (14.1)
Jola	143 (5.6)	264 (15.1)	407 (9.4)
Serahule	343 (13.4)	40 (2.3)	383 (8.9)
^+^Others	162 (6.3)	215 (12.3)	377 (8.8)

*The others are retirees

^+^ The Others include Aku, Serere, Manjago, Jahanka

The study population mean scores (standard deviations) for knowledge, attitude and practice towards TB are 8(±4), 3 (±0.9) and 1.8(±0.5) respectively.

### Knowledge about TB

Overall, most participants indicated they had heard about TB (Table 2), however TB awareness was 5% (95% CI: 2.7–7.3) higher in rural compared to urban residents and this was significantly different (*p* < 0.001).

**Table 2:** TB knowledge of the respondents stratified by area of residence

Variable	Rural residence (***N*** = 2558)	Urban residence (***N*** = 1751)	Difference in proportions	Total (***N*** = 4309)	
	**N (%)**	**N (%)**	**% (95% CI)**	**N (%)**	***P*** **value**
*Have you heard about TB?	2199 (86.0)	1418 (81.0)	5 (2.7, 7.3)	3617 (83.9)	< 0.001
*How serious the respondent regarded TB					
Life threatening	2059 (80.5)	1386 (79.2)	1.3 (−1.1, 3.8)	3445 (80.0)	0.281
Not very serious/threatening	224 (8.8)	209 (12.0)	- 3.1 (−5.1, −1.3)	433 (10.0)	0.001
Don’t know	275 (10.8)	156 (8.9)	1.8 (0.1, 3.6)	431 (10.0)	0.048
*Common TB symptoms and signs					
Cough that lasts for 2–3 weeks	1840 (71.9)	1303 (74.4)	- 2.5 (− 5.2, 0.2)	3143 (72.9)	0.072
Bloody sputum	1597 (62.4)	1142 (65.2)	- 2.8 (−5.7, 0.1)	2739 (63.6)	0.062
Weight loss	1686 (65.9)	1197 (68.4)	- 2.5 (−5.3, 0.4)	2883 (67.0)	0.093
Fever	1541 (60.2)	1021 (58.3)	1.9 (−1.1, 4.9)	2562 (59.5)	0.204
Chest pain	1674 (65.4)	1169 (66.8)	−1.3 (−4.2, 1.6)	2843 (66.0)	0.369
Shortness of breath	1571 (61.4)	1085 (62.0)	- 0.6 (−3.5, 2.4)	2656 (61.6)	0.716
*Mode of TB transmission					
Through air when a person with TB cough or sneezes	1864 (72.9)	1357 (77.5)	- 4.6 (−7.2, −2.0)	3221 (74.8)	0.001
By living with a TB patient	1149 (44.9)	834 (47.6)	- 2.7 (−5.7, 0.3)	1983 (46.0)	0.079
By sharing dishes, plates, cups and spoons	1830 (71.5)	1256 (71.7)	- 0.2 (−2.9, 2.5)	3086 (71.6)	0.892
Through smoking cigarettes or drinking alcohol	1472 (57.5)	972 (55.5)	2.0 (−0.1, 0.05)	2444 (56.7)	0.186
*Mode of prevention					
Covering mouth and nose when coughing or sneezing	1795 (70.2)	1300 (74.2)	- 4.0 (−6.8, −1.4)	3095 (71.8)	0.004
Avoid sharing dishes, spoons and cups	1794 (70.1)	1253 (71.6)	−1.4 (−4.2, 1.3)	3047 (70.7)	0.312
Avoiding people who appear to have TB	1104 (43.1)	713 (40.7)	2.4 (−0.6, 5.4)	1817 (42.2)	0.111
Through good nutrition	1059 (41.4)	613 (35.0)	6.4 (3.5, 9.3)	1672 (38.8)	< 0.001
By praying and fasting	971 (38.0)	511 (29.2)	8.8 (5.9, 11.6)	1482 (34.4)	< 0.001
Avoid handshakes	364 (14.2)	238 (13.6)	0.6 (−1.5, 2.7)	602 (14.0)	0.553
**Can TB be cured?**					
Yes	2203 (86.1)	1607 (91.8)	- 5.7 (−7.5, − 3.8)	3810 (88.4)	< 0.001
No	43 (1.7)	12 (0.7)	1.0 (0.4, 1.6)	55 (1.3)	0.004
Don’t know	312 (12.2)	132 (7.5)	4.7 (2.9, 6.4)	444 (10.3)	< 0.001
*How can someone with TB be cured? (Rural ***N*** **= 2203, Urban** ***N*** **= 1607, Total** ***N*** **= 3810)**					
Drugs specifically for TB	2132 (96.8)	1550 (96.5)	0.3 (− 0.8, 1.5)	3682 (96.6)	0.583
Herbal remedies	225 (10.2)	161 (10.0)	0.2 (−1.7, 2.1)	386 (10.1)	0.844
Praying and fasting	77 (3.5)	60 (3.7)	−0.2 (−1.4, 1.0)	137 (3.6)	0.696
Good nutrition	30 (1.4)	18 (1.1)	0.2 (−0.5, 0.9)	40 (1.3)	0.509
**Cost of TB treatment in the country?**					
It is free	932 (36.4)	782 (44.7)	- 8.2 (−11.2, − 5.2)	1714 (39.8)	< 0.001
Pay some fee	458 (17.9)	241 (13.8)	4.1 (1.9, 6.3)	699 (16.2)	< 0.001
Don’t know	1168 (45.7)	728 (41.6)	4.1 (1.1, 7.1)	1896 (44.0)	0.008
**Overall TB Knowledge**					
Poor	882 (34.5)	544 (31.1)	3.4 (0.6, 6.3)	1426 (33.1)	0.019
Good	1676 (65.5)	1207 (68.9)	- 3.4 (− 6.3, − 0.6)	2883 (66.9)	0.019

*Only the “Yes” responses tabulated

TB was considered a very serious or life-threatening condition by similar numbers of urban and rural dwellers. The 3 correct TB symptoms most commonly given by participants were long-lasting cough (72.9%), weight loss (67%) and chest pain (66%) and these responses did not differ by area of residence (see Table 2).

Overall, about three-quarters of participants correctly identified the airborne route as the mode of TB transmission. Although significantly more urban than rural residents correctly associated TB transmission with the airborne route, similar proportions of rural and urban-based participants also erroneously attributed a role to sharing cutlery and crockery in TB transmission (Table 2). With respect to preventing TB transmission, ~ 72% overall, and significantly more urban than rural-based participants correctly identified covering the mouth or nose during coughs and sneezes as useful for preventing TB transmission (Table 2). However, over 40% of all participants considered avoiding those perceived as having TB as a means of preventing transmission and this response did not vary by residence. Furthermore, over a third of all participants and predominantly more rural dwellers identified roles for good nutrition and religion (through praying and fasting) in the prevention of TB.

The majority (88.4%) of the participants understood TB was curable with a significant urban preponderance. Out of this proportion, almost everyone (96.6%) had the understanding that it can be cured by taking drugs specifically for TB while a minor proportion said herbal remedies, praying and fasting, and good nutrition are ways of TB treatment. Compared to the understanding that TB can be cured, about 40% of participants and mainly urban dwellers were aware TB treatment was free in the country (Table 2).

Among those who had knowledge of TB (Table 3), similar proportions of rural and urban resident participants first heard about TB from family, friends, neighbours and colleagues. In contrast to urban areas where television was an important source of TB awareness, the radio played a bigger role in rural communities. A significantly higher proportion of participants in the older age category i.e. ≥35 years acquired their TB knowledge from the radio compared to younger participants (15 to 34 year). As expected for the younger age category (15–34 years), TV was a more important source of TB knowledge than for their older counterparts (Table 3).

**Table 3:** Source participants first heard about TB based on residence, gender and age grouping

Variable	N (%)	N (%)	% (95% CI)	N (%)	***P*** value
**Residence**	**Rural (*N*** **= 2199)**	**Urban (*N*** **= 1418)**	**Difference in proportions**	**Total (*N*** **= 3617)**	
Family, friends and neighbours	864 (39.3)	560 (39.5)	- 0.2 (− 3.5, 3.1)	1424 (39.4)	0.22
Radio	917 (41.7)	460 (32.4)	9.3 (6.1, 12.5)	1377 (38.0)	< 0.001
TV	159 (7.2)	260 (18.3)	−11.1 (− 13.4, − 8.8)	419 (11.6)	< 0.001
*Others	259 (11.8)	138 (9.7)	2.1 (− 0.0, 4.1)	397 (11.0)	0.06
**Gender**	**Female (*N*** **= 2168)**	**Male (*N*** **= 1449)**	**Difference in proportions**	**Total (N = 3617)**	
Family, friends and neighbours	823 (38.0)	601 (41.5)	- 3.5 (− 6.8, − 0.3)	1424 (39.4)	0.03
Radio	831 (38.3)	546 (37.7)	0.6 (− 2.6, 3.9)	1377 (38.0)	0.69
TV	261 (12.0)	158 (10.9)	1.1 (−1.0, 3.2)	419 (11.6)	0.30
*Others	253 (11.7)	144 (9.9)	1.8 (− 0.3, 3.8)	397 (11.0)	0.10
**Age grouping**	**15–34 years (*N*** **= 2237)**	**≥35 years (*N*** **= 1380)**	**Difference in proportions**	**Total (N = 3617)**	
Family, friends and neighbours	874 (39.1)	550 (39.9)	- 0.8 (− 4.1, 2.5)	1424 (39.4)	0.64
Radio	808 (36.1)	569 (41.2)	- 5.1 (− 8.4, −1.8)	1377 (38.0)	0.002
TV	299 (13.4)	120 (8.7)	4.7 (2.6, 6.7)	419 (11.6)	< 0.001
*Others	256 (11.4)	141 (10.2)	1.2 (− 0.8, 3.3)	397 (11.0)	0.25

*This include teachers, health workers, print media, religious leaders

### Overall knowledge

Given their scores, two-thirds of all participants had overall good knowledge of TB and urban residents significantly had more knowledge than their rural counterparts (see Table 2).

We assessed a range of exposures for associations with overall TB knowledge as shown in Table 4. After controlling for other variables in the model, age (35 – 64 yrs), marital status, education, being employed, a favourable attitude towards TB and good practice with respect to TB were all predictors of good TB knowledge. With education, there was a dose-response relationship as the biggest effect was seen with education to secondary level and above. There was no evidence interaction between residence and age or educational status nor between marital status and age or educational status or residence (data not shown).

**Table 4:** Factors associated with good knowledge among the respondents

Variable (***n*** = 4309)	Unadjusted			Adjusted for all the variables		
	OR	95% CI	***P***-value	OR	95% CI	***P***-value
**Sex**						
Female^+^	1.00			1.00		
Male	1.33	1.15–1.53	< 0.001	1.04	0.86–1.26	0.687
**Age group**						
15 – 34 yrs.^+^	1.00			1.00		
35 – 64 yrs	1.42	1.21–1.65	< 0.001	1.40	1.15–1.69	**0.001**
≥ 65 yrs	1.04	0.76–1.43	0.806	1.19	0.83–1.71	0.337
**Marital status**						
Single^+^	1.00			1.00		
married	1.38	1.19–1.60	0.001	1.54	1.27–1.88	**< 0.001**
Once married*	0.97	0.68–1.37	0.858	1.31	0.86–1.98	0.210
**Educational status**						
illiterate^+^	1.00			1.00		
Read English/Arabic	1.25	1.02–1.51	0.027	1.35	1.08–1.67	**0.007**
Primary/Incomplete Sec. school	1.46	1.20–1.76	< 0.001	1.97	1.57–2.46	**< 0.001**
Secondary school & above	2.70	2.14–3.41	< 0.001	3.67	2.79–4.84	**< 0.001**
**Employment status**						
unemployed^+^	1.00			1.00		
Employed	1.59	1.37–1.84	< 0.001	1.42	1.19–1.70	**< 0.001**
**Residence**						
Rural^+^	1.00			1.00		
Urban	1.21	0.76–1.91	0.421	0.95	0.53–1.71	0.874
**Attitude towards TB**						
Unfavorable^+^	1.00			1.00		
Favourable	2.66	2.21–3.20	< 0.001	2.30	1.90–2.78	**< 0.001**
**Practice towards TB**						
Poor^+^	1.00			1.00		
Good	2.38	1.87–3.03	< 0.001	2.03	1.58–2.60	**< 0.001**

^+^This is the reference category

^*^This comprises of widow(er) and separated/divorced

### Attitudes and practice towards TB

As shown in Table 5, just over half of all participants thought they could get TB and significantly more urban dwellers recognised this possibility than the rural ones. With respect to their range of feelings if diagnosed with TB, majority of rural compared to urban residents identified with negative feelings of fear, surprise, shame, embarrassment, sadness and hopelessness. Over three-quarters of participants also indicated they would be happy if diagnosed with TB because the underlying cause of their ill-health would have been identified and can be treated. However, this view was held by predominantly more urban participants compared to the rural (see Table 5).

**Table 5:** TB attitudes of respondents stratified by area of residence

Variable	Rural residence (***N*** = 2558)	Urban Residence (***N*** = 1751)	Difference in proportions	Total (***N*** = 4309)	***p***-value
	***N*** (%)	***N*** (%)	% (95% CI)	***N*** (%)	
*Do you think you can get TB?	1370 (53.6)	1103 (63.0)	- 9.4 (−12.4, − 6.5)	2473 (57.4)	**< 0.001**
*How would you feel if you were found to have TB?					
Fear	1549 (60.6)	868 (49.6)	11.0 (8.0, 14.0)	2417 (56.1)	**< 0.001**
Surprise	1700 (66.5)	1015 (58.0)	8.5 (5.5, 11.4)	2715 (63.0)	**< 0.001**
Shame	851 (33.3)	440 (25.1)	8.1 (5.4, 10.9)	1291 (30.0)	**< 0.001**
Embarrassment	842 (32.9)	441 (25.2)	7.7 (5.0, 10.5)	1283 (29.8)	**< 0.001**
Sadness	1651 (64.5)	1017 (58.1)	6.5 (3.5, 9.4)	2668 (61.9)	**< 0.001**
Hopelessness	649 (25.4)	384 (21.9)	3.4 (0.9, 6.0)	1033 (24.0)	**0.009**
Happiness	1903 (74.4)	1383 (79.0)	- 4.6 (− 7.1, − 2.0)	3286 (76.3)	**0.001**
*Who would you talk to about your illness if you had TB?					
Health workers	2440 (95.4)	1667 (95.2)	0.2 (−1.1, 1.5)	4107 (95.3)	0.779
Spouse	2343 (91.6)	1562 (89.2)	2.4 (0.6, 4.2)	3905 (90.6)	**0.008**
Parents	2329 (91.0)	1572 (89.8)	1.3 (− 0.5, 3.1)	3901 (90.5)	0.162
Children	2234 (87.3)	1465 (83.7)	3.7 (1.5, 5.8)	3699 (85.8)	**0.001**
Landlord/Household head	2230 (87.2)	1426 (81.4)	5.7 (3.5, 8.0)	3656 (84.9)	**< 0.001**
Close friend	2032 (79.4)	1368 (78.1)	1.3 (−1.2, 3.8)	3400 (78.9)	0.300
**How do you feel about people with TB?**					
I try to help	1233 (48.2)	948 (54.1)	- 5.9 (− 9.0, − 2.9)	2181 (50.6)	**< 0.001**
I stay away from them	1186 (46.4)	710 (40.6)	5.8 (2.8, 8.8)	1896 (44.0)	**< 0.001**
Treat them like I treat other people	139 (5.4)	93 (5.3)	0.1 (− 1.2, 1.5)	232 (5.4)	0.861
**Overall attitude**					
Unfavourable	2040 (79.7)	1280 (73.1)	6.6 (4.1, 9.2)	3320 (77.0)	**< 0.001**
favourable	518 (20.3)	471 (26.9)	- 6.6 (− 9.2, − 4.1)	989 (23.0)	**< 0.001**

*Only the “Yes” responses tabulated

In addition to speaking to their relations, household head and close friends following a diagnosis of TB, majority of participants and similar numbers by residence indicated willingness to speak to health workers. While many more rural than urban participants indicated they would speak to spouse, children and household heads about their diagnosis, fewer urban participants were likely to discuss their diagnosis with their landlord or household head. When asked about how they feel about persons with TB, a little under half of all participants expressed a preference for staying away from them rather than providing support. As shown in Table 5, this preference for withdrawing from TB patients was expressed in more rural than urban residents.

There was near universal preference for visiting a health centre among participants if they felt they had TB symptoms and indication of good health seeking behaviour although with a slight but significant urban preponderance. Most (84.2%) of the participants reported that they would go immediately if they develop TB symptoms. There was no significant difference by sex in the participant’s preference for visiting a health centre (data not shown). In addition, inhabitants of rural parts of the country were more likely to consider self-treatment and consult traditional healers and religious leaders as indicated in Table 6.

**Table 6:** TB practice of respondents stratified by area of residence

*What would you do if you had TB symptoms?					
Go to the health facility	2440 (95.4)	1717 (98.1)	- 2.7 (− 3.7, − 1.6)	4157 (96.5)	**< 0.001**
Go to pharmacy	167 (6.5)	110 (6.3)	0.2 (− 1.2, 1.7)	277 (6.4)	0.746
Go to traditional healer	305 (11.9)	116 (6.6)	5.3 (3.6, 7.0)	421 (9.8)	**< 0.001**
Go to religious leader	83 (3.2)	29 (1.7)	1.6 (0.7, 2.5)	112 (2.6)	**0.001**
Pursue self-treatment	88 (3.4)	30 (1.7)	1.7 (0.8, 2.7)	118 (2.7)	**0.001**
**At what point will you go to the health facility if you develop TB symptoms?**					
Will go immediately	2092 (81.8)	1535 (87.7)	- 5.9 (− 8.0, − 3.7)	3627 (84.2)	**< 0.001**
Will not go at all	102 (4.0)	24 (1.4)	2.6 (1.7, 3.6)	126 (2.9)	**< 0.001**
**Others	364 (14.2)	192 (11.0)	3.3 (1.3, 5.3)	556 (12.9)	**0.002**
**Overall practice**					
Poor	477 (18.6)	225 (12.8)	5.8 (3.6, 8.0)	702 (16.3)	**< 0.001**
Good	2081 (81.4)	1526 (87.2)	- 5.8 (− 8.0, − 3.6)	3607 (83.7)	**< 0.001**

*Only the “Yes” responses tabulated

**if symptoms don’t get worse/when self-treatment fails/wait for 3 to 4 weeks

### Overall attitude and practice

Most participants in this survey had an unfavourable attitude towards TB and this attitude was exhibited mostly by rural residents. In the multivariable analyses, favourable attitude towards TB was associated with being male, married, having at least primary level education, being employed, having good TB knowledge and good practice towards TB (Table 7). However, the underlying attitude was different if participants were directly affected by TB. The majority of participants had good practice regarding TB if they were personally affected, and this was significantly more in urban compared to rural residents. The key predictors of good practice for TB were being married, having at least primary school level education, having a job, good TB knowledge and favourable attitude towards TB (Table 8).

**Table 7:** Factors associated with favourable attitudes among the respondents

Variable (***n*** = 4309)	Unadjusted			Adjusted for all the variables		
	OR	95% CI	***P***-value	OR	95% CI	***P***-value
**Sex**						
Female^+^	1.00			1.00		
Male	1.45	1.24–1.70	< 0.001	1.25	1.03–1.53	0.028
**Age group**						
15 – 34 yrs.^+^	1.00			1.00		
35 – 64 yrs	1.34	1.13–1.58	0.001	1.12	0.91–1.37	0.296
≥ 65 yrs	1.14	0.79–1.64	0.479	1.23	0.82–1.85	0.311
**Marital status**						
Single^+^	1.00			1.00		
Married	1.51	1.28–1.79	< 0.001	1.67	1.34–2.07	< 0.001
Once married	0.74	0.48–1.16	0.193	0.94	0.56–1.55	0.799
**Educational status**						
Illiterate^+^	1.00			1.00		
Primary/Incomplete Sec. school	1.21	0.98–1.51	0.075	1.39	1.09–1.77	0.008
Read English/Arabic	1.19	0.96–1.49	0.114	1.09	0.86–1.39	0.475
Sec. school & above	2.01	1.61–2.52	< 0.001	2.04	1.57–2.65	< 0.001
**Employment status**						
Unemployed^+^	1.00			1.00		
Employed	1.66	1.42–1.95	< 0.001	1.30	1.07–1.57	0.008
**Residence**						
Rural^+^	1.00			1.00		
Urban	1.63	1.00–2.66	0.049	1.48	0.87–2.50	0.144
**Knowledge of TB**						
Poor^+^	1.00			1.00		
Good	2.65	2.20–3.19	< 0.001	2.21	1.83–2.68	< 0.001
**Practice towards TB**						
Poor^+^	1.00			1.00		
Good	3.27	2.33–4.59	< 0.001	2.80	1.97–3.97	< 0.001

^+^This is the reference category

**Table 8:** Factors associated with good practice among the respondents

Variable (n = 4309)	Unadjusted			Adjusted for all the variables		
	OR	95% CI	***P***-value	OR	95% CI	***P***-value
**Sex**						
Female^+^	1.00			–		
Male	0.99	0.82–1.19	0.900	**–**	**–**	**–**
**Age group**						
15 – 34 yrs.^+^	1.00			–		
35 – 64 yrs	1.07	0.87–1.31	0.518	**–**	**–**	**–**
≥ 65 yrs	0.83	0.56–1.23	0.358	**–**	**–**	**–**
**Marital status**						
Unmarried^+^	1.00			1.00		
Married	1.30	1.06–1.59	0.013	1.63	1.28–2.08	< 0.001
Once married	0.92	0.60–1.41	0.716	1.34	0.84–2.13	0.220
**Educational status**						
Illiterate^+^	1.00			1.00		
Primary/Incomplete Sec. school	1.52	1.18–1.94	0.001	1.82	1.37–2.42	< 0.001
Read English/Arabic	1.16	0.88–1.51	0.292	1.17	0.88–1.56	0.282
Sec. school & above	4.05	2.70–6.05	< 0.001	4.08	2.65–6.23	< 0.001
**Employment status**						
Unemployed^+^	1.00			1.00		
Employed	1.38	1.13–1.68	0.001	1.25	1.01–1.54	0.039
**Residence**						
Rural^+^	1.00			–		
Urban	1.93	0.78–4.76	0.152	**–**	**–**	**–**
**Knowledge of TB**						
Poor^+^	1.00			1.00		
Good	2.62	2.05–3.34	< 0.001	2.08	1.62–2.67	< 0.001
**Attitude towards TB**						
Unfavorable^+^	1.00			1.00		
Favourable	3.09	2.19–4.36	< 0.001	2.26	1.58–3.23	< 0.001

^+^This is the reference category

## Discussion

From 2010 to 2019, there were 16 nationwide TB prevalence surveys in African countries including The Gambia [1]. However, only Gambia of all these countries seems to have also conducted a TB knowledge, attitudes and practice concurrently. By studying a nationally representative population in the context of a nationwide TB prevalence survey, we were able to investigate the knowledge, attitude and practice with respect to TB on a national level for the first time in The Gambia.

Of the 80% of our population who had some knowledge of TB, only two-thirds had good or appropriate knowledge, and a significantly higher proportion of them were urban residents. Good TB knowledge in The Gambia was associated with age, marital status, education, employment, a good TB attitude and practice. These findings are consistent with those from other studies despite our lower prevalence of participants with appropriate knowledge [30–35].

We observed a discordance between responses regarding the mode of TB transmission. On one hand, majority of the participants correctly identified the airborne route as the means by which TB is transmitted but up to two-thirds also believe TB transmission was still possible if cutlery and crockery are shared with persons with TB, and that avoiding this was a means of TB prevention. This suggests that while ACSM and other interventions might have introduced the population to the concept of airborne transmission for TB, more work is required to change their core beliefs and practice regarding TB transmission. Given the wider access to radio in ours and other similar settings, [36, 37] it was not surprising to find radio was the main source of TB knowledge in rural areas while TV played a bigger role in urban areas in The Gambia. However, it was not a common source of TB information as observed in other studies [15, 38]. Similar to our study, others [34] have also found that communities acquire TB knowledge from their family, friends, and colleagues. These suggest contextualised interventions via the radio and/or community-based may be an effective way in reaching out to a lot of people to improve and consolidate their TB knowledge. There was no association between TB knowledge and sex as reported by other investigators [15, 36], however, some other studies have reported an association [14, 33, 35, 39].

We found a population with a predominantly negative attitude to TB including high levels of stigmatising behaviour, especially in rural residents which may contribute to the gaps in TB detection and treatment seen in The Gambia and other African TB prevalence surveys. It is our experience from TB case-contact research in urban Gambia [40] that newly diagnosed TB patients are frequently isolated in their households. In addition, they were often allocated their own kitchen utensils within their households. This practice is no doubt the consequence of erroneous belief that TB transmission is possible if cutlery and crockery are shared with persons with TB and contributes to the level of stigmatisation observed in this study. Furthermore, this stigma is also fuelled by the negative attitudes of fear, avoidance, surprise, shame and sadness that participants indicated they would feel if ever diagnosed with TB. When considered alongside the poor TB knowledge exhibited by 3 out of 10 persons in The Gambia especially in rural areas, this is a considerable challenge that needs to be tackled head on as part of TB control efforts [41, 42] especially in a country with mostly rural dwelling population [26]. Male sex, marriage, at least primary education, employment, a good knowledge and practice of TB were predictive of more favourable attitudes to TB. However, the association of male sex with a positive attitude towards TB is unexpected given the Gambian TB prevalence survey [3] just like others [4, 43, 44] and in concordance with global TB epidemiology shows majority of TB cases are males [1]. This may be closely related to their health-seeking behaviour when they have TB-like symptoms [9].

Most of our participants had good health seeking behaviour or practice regarding TB. In contrast, most of the TB cases in the prevalence survey were previously undiagnosed in the community and were not on treatment [3]. This disparity between health seeking behaviour and case detection in the community underlies the importance of ensuring health centres are adequately equipped, and health workers are adequately trained to, facilitate early diagnosis and treatment of TB. These will help prevent missed opportunities for TB diagnosis and reduce the spread of TB and possibly associated stigma [1, 18, 38, 44, 45]. It is possible that reported health-seeking behaviours might be different from actual actions which may also explain the discordance between a favourable attitude to TB and the preponderance of undiagnosed male TB cases during the TB prevalence survey in Gambia and elsewhere.

The general predictors of good knowledge, attitude and health-seeking behaviour regarding TB in our study are being married, having some form of education and being employed. This was also observed by Luba et al. [33] while other studies [13–15] also found similar relationships with education and employment status. The participants in this study who were married were more likely to be in the older (35–64 years) age group that had a very good knowledge of TB and good TB knowledge was significantly associated with favourable attitude and good health-seeking behaviour towards TB. However, we did not find any interaction between marital status and age or educational status in our final model. This suggests that improvement in TB awareness campaign activities irrespective of educational background of a population is important for TB control efforts [31, 32, 35, 46].

Our large sample size and a nationally representative population are main strengths of this study. Given the cross-sectional design, our inference regarding the directionality of the ‘predictors’ of good knowledge, attitude and practice is somewhat limited and requires further investigation. Beyond the factors explored here that are co-determinants of health-seeking behaviour, factors directly related to health care system also contribute to delays in TB diagnosis and treatment but these were not the focus of this study. Nonetheless, well informed communities in addition to identification of other structural contributors to delays in seeking TB care have been identified as important for TB control [20, 21, 47–49]. Our study provides valuable insights regarding societal knowledge of TB, exposes the significant stigma in the community and identifies some areas requiring intervention by the national TB programme and other stakeholders in the fight to control TB in The Gambia and similar settings.

## Conclusions

In conclusion, whilst the majority of participants had good health-seeking practice, there was still significant stigma associated with TB as well as knowledge gaps on transmission, prevention and cost of treatment of TB. Focused community-based campaigns to educate people on TB are required as part of community engagement for TB control in the NLTP’s ACSM strategy. In addition, the use of radio and television tailored to deliver TB education to rural and urban dwellers should complement the community engagement strategies for a wider reach. Training and continued re-training of health care workers especially community and village health workers to educate the public on TB at every opportunity they get are also critical to all of the efforts outlined above.

## Supplementary information

**Additional file 1.** Questionnaire for TB KAP Study – contains 29 questions used for the survey about TB knowledge, attitude and care-seeking behaviour as well as their source of information regarding TB.

## Data Availability

The data that supports the findings in this study can be made available through contacting the corresponding author under reasonable request and with permission from the director of The Medical Research Council Unit, the Gambia (MRCG).

## Abbreviations

ACSM: Advocacy, Communication and Social Mobilization
GAMSTEP: Gambian Survey of Tuberculosis Prevalence
KAP: Knowledge, Attitude and Practice
MRCG: Medical Research Council Unit, The Gambia
NLTP: National Leprosy and Tuberculosis Progamme
TB: Pulmonary Tuberculosis
