# Supplementary material for: Knowledge, attitude and practice towards tuberculosis in Gambia: a nation-wide cross-sectional survey
Source: BMC Public Health. 2020 Oct 17;20:1566. doi: 10.1186/s12889-020-09685-3 (PMC7568354; doi:10.1186/s12889-020-09685-3)
Supplement: Supplementary file 1 — Additional file 1. Questionnaire for TB KAP Study – contains 29 questions used for the survey about TB knowledge, attitude and care-seeking behaviour as well as their source of information regarding TB. [file 12889_2020_9685_MOESM1_ESM.pdf]

National Leprosy and Tuberculosis Programme

The Gambian Survey of TB Prevalence (GAMSTEP) SCC 1232

Questionnaire for TB KAP Study-Form 5

MRC The Gambia Unit

(To be filled in by Interviewer)

1. **Individual Survey No.** ☐☐☐ ☐☐☐ ☐☐☐
2. **Name:** ____
3. **Age**: |__|__|years 4. **Sex**: ☐ Male ☐ Female
4. **Interviewer code**|__|__| *(initials)*
5. **Date:** |__|__|/|__|__|/|__|__|
   dd mm y y

DO NOT READ RESPONSES UNLESS INDICATED IN THE DIRECTIONS

Health-seeking behaviour

1. Where do you normally go when you are sick and need treatment? **(Select only one best answer)**

   ☐1. Private clinic
   ☐2. Government health centre/dispensary/clinic or hospital
   ☐3. Traditional healer/Marabout
   ☐4. Health care facilities run by nongovernmental organization (NGO) or religious body
   ☐5. Other: specify____

2. How often did you seek care at the following in the last 1 year? **(Choose one answer option for each of Q2a-Q2d)**

   a. Government Health Centre/dispensary

   ☐1. Once or twice
   ☐2. 3-5 times
   ☐3. More than 5 times
   ☐4. Never in past 1 year
   ☐5. Other: specify ____

   b. Government Hospital

   ☐1. Once or twice
   ☐2. 3-5 times
   ☐3. More than 5 times
   ☐4. Never in past 1 year
   ☐5. Other: specify____

   c. Traditional healer/marabout

   ☐1. Once or twice
   ☐2. 3-5 times
   ☐3. More than 5 times
   ☐4. Never in past 1 year
   ☐5. Other: specify ____

d. Health care facilities run by nongovernmental organization (NGO) or religious body

☐1. Once or twice
☐2. 3-5 times
☐3. More than 5 times
☐4. Never in past 1 year
☐5. Other: specify ____

TB knowledge and awareness

3. Where did you first learn about tuberculosis or TB? Did you learn about TB through...? **(Prompt the respondent by listing and going through the entire list and tick one correct answer)**

☐1. Newspapers and magazines
☐2. Radio
☐3. TV
☐4. Billboards
☐5. Brochures, posters and other printed materials
☐6. Health workers
☐7. Family, friends, neighbours and colleagues
☐8. Religious leaders
☐9. Teachers
☐10. Other (please explain):
____
☐11. I don't know [or don't remember]

4. In your opinion, if you thought you had TB, how serious would you think it is and how soon would you seek health care services? **(Prompt the respondent by going through all the options then, Check one answer.)**

☐1. Very serious/life threatening requiring immediate attention (not later than 1day)
☐2. Not life threatening or risky so can be dealt with in 2-4 days
☐3. Not very serious and can be dealt with in 5 days or more
☐4. I don't know

5. How would you rate TB as a problem in your community? **(Check one.)**

☐1. It is a big problem
☐2. It exists but is not too much of a problem
☐3. It is not a problem at all
☐ 4. I do not know

6. How would you rate TB as a problem in The Gambia as a whole? **(Check one.)**

☐1. Very big problem
☐2. It exists but is not too much of a problem
☐3. It is not a problem at all
☐4. I don't know

7. Which of the following do you think as symptoms and/or signs of TB? (NB: DK=Don't know)

   a. Rash ☐Yes; ☐No; ☐ DK
   b. Severe headache ☐Yes; ☐No; ☐ DK
   c. Cough that lasts for 2-3 weeks or more ☐Yes; ☐No; ☐ DK
   d. Vomiting or feeling like vomiting ☐Yes; ☐No; ☐ DK
   e. Coughing up blood ☐Yes; ☐No; ☐ DK
   f. Weight loss ☐Yes; ☐No; ☐ DK
   g. Yellow eyes ☐Yes; ☐No; ☐ DK
   h. Back pain ☐Yes; ☐No; ☐ DK
   i. Fever ☐Yes; ☐No; ☐ DK
   j. Chest pain ☐Yes; ☐No ; ☐ DK
   k. Shortness of breath ☐Yes; ☐No; ☐ DK
   l. Tiredness or easy fatigability ☐Yes; ☐No; ☐ DK
   m. Other: specify____

8. In what ways can a person get TB? **(Please prompt for answers)** (NB: DK=Don't know)

   a. By sharing things such as biros, hoes, cutlasses, other work objects ☐Yes; ☐No; ☐ DK
   b. Through handshakes ☐Yes; ☐No; ☐ DK
   c. Through the air when a person with TB coughs or sneezes ☐Yes; ☐No; ☐ DK
   d. By living in the same country/community or neighbourhood with a person with TB → ☐Yes; ☐No; ☐ DK
   e. Through sharing dishes, plates cups and spoons ☐Yes; ☐No; ☐ DK
   f. Through smoking cigarettes or drinking alcohol ☐Yes; ☐No; ☐ DK
   g. Through touching items in public places (doorknobs, walls, tables, chairs) ☐Yes; ☐No; ☐ DK
   h. Others (please explain):____

9. In what ways can a person avoid getting TB? **(Please prompt for answers and tick Yes or NO for each option)** (NB: DK=Don't know)

   a. Avoid shaking hands ☐Yes; ☐No; ☐ DK
   b. Covering mouth and nose when coughing or sneezing ☐Yes; ☐No; ☐ DK
   c. Avoid sharing dishes, spoons and cups ☐Yes; ☐No; ☐ DK
   d. Washing hands after touching items in public places ☐Yes; ☐No; ☐ DK
   e. Closing windows at home ☐Yes; ☐No; ☐ DK
   f. Through good nutrition/eating well ☐Yes; ☐No; ☐ DK
   g. By praying and/or fasting ☐Yes; ☐No; ☐ DK
   h. By avoiding people who appear like they have TB ☐Yes; ☐No; ☐ DK
   i. Other (please explain____)

10. In your opinion, who can be infected with TB? **(Please prompt for answers and tick Yes or NO for each option)** (NB: DK=Don't know)

   a. Anybody ☐Yes; ☐No; ☐ DK

b. Only poor people ☐Yes; ☐No; ☐ DK
c. Only people who smoke cigarettes ☐Yes; ☐No; ☐ DK
d. Only people who drink a lot of alcohol ☐Yes; ☐No; ☐ DK
e. Only people who use hard drugs ☐Yes; ☐No; ☐ DK
f. Only people living with HIV/AIDS ☐Yes; ☐No; ☐ DK
g. Only people who have been in prison ☐Yes; ☐No; ☐ DK
h. Other (please explain):____)

11. Can TB be cured? IF NO, GO TO QUESTION 13

☐1. Yes
☐2. No
☐3. I don't know

12. How can someone with TB be cured? (**Please prompt for answers and tick Yes or NO for each option**)

a. Marabout/Herbal remedies ☐Yes; ☐No
b. Home rest without medicine ☐Yes; ☐No
c. Eating good food ☐Yes; ☐No
d. Praying and/or fasting ☐Yes; ☐No
e. Drugs specifically for TB ☐Yes; ☐No
f. Other drugs given at the health centre ☐Yes; ☐No
g. Do not know ☐Yes; ☐No
h. Other: (specify)____

TB Attitudes and Care-seeking Behaviour

13. Do you think you can get TB? **(Ask respondent to please explain his/her answer.)**

☐1.Yes (because…)____
____
____

☐2.No(because…)____
____
____

☐3. I don't know

14. How would you feel if you were found to have TB? **(Please prompt for answers and tick Yes or NO for each option)**

a. Fear ☐Yes; ☐No
b. Surprise ☐Yes; ☐No
c. Shame ☐Yes; ☐No
d. Embarrassment ☐Yes; ☐No

e. Sadness ☐Yes; ☐No
f. Hopelessness ☐Yes; ☐No
g. Happiness (because my problem has been identified and can be treated) ☐Yes; ☐No
h. Other(specify):______________________________
i. I don't know ☐Yes; ☐No

15. Who would you talk to about your illness if you had TB? **(Please prompt for answers and tick Yes or NO for each option)**
   a. Landlord ☐Yes; ☐No
   b. Health care worker-doctor, nurse, CHN, VHW ☐Yes; ☐No
   c. Spouse (participant's wife or husband) ☐Yes; ☐No
   d. Neighbour ☐Yes; ☐No
   e. Parents ☐Yes; ☐No
   f. Child(ren) ☐Yes; ☐No
   g. Other family member ☐Yes; ☐No
   h. Close friend ☐ Yes; ☐No
   i. No one ☐ Yes; ☐No
   j. Other: specify______________________________

16. What would you do if you had the following symptoms- cough for 2 weeks or more, night sweats, fever and some weight loss? **(Please prompt for answers and tick Yes or NO for each option)**
   a. Go to health facility ☐Yes; ☐No
   b. Nothing or wait for symptoms to progress some more ☐Yes; ☐No
   c. Go to pharmacy ☐Yes; ☐No
   d. Go to traditional healer/Marabout ☐Yes; ☐No
   e. Go to religious leader ☐Yes; ☐No
   f. Pursue other self-treatment options (herbs, etc.) ☐Yes; ☐No
   g. Stay at home and wait for the body to heal itself of the TB ☐Yes; ☐No
   h. Other:______________________________
   i. I don't know ☐Yes; ☐No

17. If you had the following symptoms, cough for 2 weeks or more, night sweats, fever and some weight loss, at what point would you go to the health facility? (**Please go through answers and tick one)**
   ☐1. I would not go to the doctor as long as symptoms don't get worse <u>**>IF YES, GOTO Q19**</u>
   ☐2. When treatment on my own does not work <u>**>IF YES, GOTO Q19**</u>
   ☐3. When symptoms that suggest TB last for 3–4 weeks <u>**>IF YES, GOTO Q19**</u>
   ☐4. I will not go to a health care facility at all. <u>**>IF YES, GOTO Q18**</u>
   ☐5. I will go immediately. <u>**>IF YES, GOTO Q19**</u>

18. Why would you not want to go to any health care facility, if you had the symptoms in Q17 above? **(Please check all that apply.)**
   ☐1. Not clear that the symptoms are serious
   ☐2. Not sure where to go
   ☐3. It will be too expensive/I cannot afford the cost

☐4. Difficulties with transportation/distance to clinic
☐5. Do not trust medical workers
☐6. Do not like attitude of medical workers
☐7. Cannot leave work (overlapping work hours with medical facility working hours)
☐8. Do not want to find out that something is really wrong
☐9. Other (please explain):____

19. How expensive do you think TB diagnosis and treatment is in this country? (**Please check one**)

☐1. It is free of charge
☐2. It is easily affordable
☐3. It is somewhat/moderately expensive
☐4. It is very expensive
☐5. Don't know

TB Attitudes and Stigma

20. Do you know people who have/had TB?

☐1. Yes
☐2. No

21. Which statement is closest to your feeling about people with TB disease? **(Read the following choices and check one answer.)**

☐1. I try to help them

☐2. I try to stay away from these people because they may infect me

☐3. I act to them as towards all other people

☐4. I want to help them but stay away from them

22. In your community, how is a person who has TB usually regarded/treated? (**Ask the question without providing any answer options but tick the category the subject's answer belongs to)**

☐1. Most people reject him or her
☐2. Their families and friends isolate them and keep them in a separate room with separate cups, plates and spoons
☐3. Most people are friendly, but they generally try to avoid him or her
☐4. The community mostly supports and helps him or her
☐5. Other (please explain):____
____
☐6. Don't know (nobody in my community has had TB)

23. Do you think that HIV positive people should be concerned about TB?

☐1. Yes > **GO TO 24a**
☐2. No > **GO TO 24b**
☐3. Don't know

24.

a. Why?
☐1. Person with HIV is more likely to develop TB
☐2. Do not know
☐3. Other: ____

b. Why not?

☐1. Person with HIV is not more likely than person without HIV to develop TB
☐2. Do not know
☐3. Other:

TB Awareness and Sources of Information

25. Do you feel well informed about TB?

☐1. Yes
☐2. No
☐3. Don't know

26. Do you wish you could get more information about TB?

☐1. Yes >**<u>GO TO Q28 and Q29</u>**
☐2. No >**<u>GO TO Q27</u>**
☐3. Don't know

27. Why do you not wish to get more information about TB?

☐1. I know enough about TB
☐2. I am not interested
☐3. I am a health care worker
☐4. It is not essential to know more about TB

28. Who would you prefer provides this information? **(Check subjects preference, one answer required)**

☐1. Health workers at the health centre
☐2. Doctor or Nurse
☐3. Community Health Nurse
☐4. Village health worker
☐5. Don't know
☐6. Anyone will do
☐7. Radio
☐8. TV
☐9. Other:____

29. How would you like this information to be delivered? **(Check subjects preference, one answer required)**

☐1. Community outreach
☐2. Newspaper
☐3. through music (including traditional dancers/dance groups)
☐4. via the radio
☐5. via the TV
☐6. by Relatives
☐7. at the mosque
☐8. Don't know
☐9. Health facility
☐10. Other:____

END OF INTERVIEW
